# CD22 regulates Aβ clearance in microglia through INPP5D signaling

**DOI:** 10.3389/fncel.2026.1886995

**Published:** 2026-07-28

**Authors:** De-Zhi Zou, Jun Li, Qiu-Shuang Long, Ruo-Qi Zhang, Bing-Lin Zhu, Jia-Ze Tan

**Affiliations:** 1 Department of Neurology, The First Affiliated Hospital of Chongqing Medical University, Chongqing, China; 2 Brain Research Center and State Key Laboratory of Trauma, Burns, and Combined Injury, The Army Medical University (Third Military Medical University), Chongqing, China; 3 Institute of Advanced Pathology Research, Jinfeng Laboratory, Chongqing, China

**Keywords:** Alzheimer's disease, Aβ clearance, CD22, INPP5D, primary microglia

## Abstract

**Introduction:**

Impaired microglial clearance of amyloid-β (Aβ) is a central driver of Alzheimer's disease (AD), yet the mechanisms governing intracellular Aβ degradation remain poorly defined.

**Methods:**

We investigated the role of CD22 in microglial Aβ processing using primary microglia, transcriptomic profiling, and hippocampal samples from AD patients and transgenic mice.

**Results:**

We identified CD22 as an Aβ-inducible signaling regulator that restrains microglial Aβ processing. CD22 expression was elevated in the hippocampus of AD patients and transgenic mice and was rapidly induced by Aβ in microglia. Functionally, *CD22* knockdown enhanced intracellular Aβ degradation without affecting initial uptake. Transcriptomic profiling identified the AD risk gene INPP5D as a key downstream effector of CD22. Loss of *CD22* suppressed INPP5D expression and promoted AKT activation, while INPP5D overexpression partially rescued the enhanced Aβ clearance phenotype. These findings define a CD22-INPP5D signaling axis that acts as a molecular brake on microglial Aβ processing. Aβ itself induces CD22 expression, revealing a feed-forward inhibitory loop in which amyloid accumulation limits its own clearance.

**Discussion:**

Collectively, these findings identify a regulatory mechanism controlling intracellular Aβ degradation and suggest the CD22-INPP5D axis as a potential therapeutic target for AD.

## 1 Introduction

Alzheimer's disease (AD) is the most common cause of dementia worldwide. It is pathologically characterized by the extracellular accumulation of amyloid-β (Aβ) plaques and intracellular neurofibrillary tangles composed of hyperphosphorylated tau (Zhang et al., 2024; Prakash et al., 2025). Accumulating evidence implicates impaired Aβ clearance, rather than overproduction alone, as a critical driver of disease initiation and progression (Coimbra et al., 2024; Takatori et al., 2025). Among the cellular mechanisms for Aβ removal, microglia, the resident immune cells of the central nervous system, serve as primary mediators of Aβ uptake and degradation (Lv et al., 2024; Ni et al., 2024; Van Olst et al., 2025). In AD, however, microglial phagocytic capacity progressively declines, contributing to amyloid accumulation and chronic neuroinflammation.

Genome-wide association studies (GWAS) have identified numerous AD risk genes that are highly expressed in microglia, underscoring the central role of immune signaling in AD pathogenesis (Bellenguez et al., 2022; Yang et al., 2023). One such gene, *INPP5D* (encoding SHIP1), produces an inositol 5′-phosphatase that negatively regulates AKT signaling downstream of immune receptors (Samuels et al., 2023). By limiting PI3K-AKT signaling, INPP5D functions as a negative regulator of microglial activation and phagocytic activity. *INPP5D* variants are linked to increased AD risk, and elevated INPP5D expression has been observed in AD brains, suggesting it may restrain microglial activation and phagocytic function (Samuels et al., 2023; Terzioglu and Young-Pearse, 2023). Despite these observations, the upstream regulators controlling INPP5D expression in microglia remain poorly defined.

CD22, a member of the sialic acid-binding immunoglobulin-like lectin (Siglec) family, is classically known as a B-cell inhibitory receptor (Brzezicka and Paulson, 2023). Recent work has detected CD22 in aging-associated microglia, where it acts as a negative regulator of phagocytosis (Pluvinage et al., 2019). CD22 blockade can restore phagocytic function in aged brains and enhance the clearance of myelin debris and amyloid-β, positioning CD22 as an immune checkpoint that modulates microglial homeostasis in neurodegeneration (Pluvinage et al., 2019; Aires et al., 2021). Previous studies have identified CD22 as an inhibitory receptor that negatively regulates microglial phagocytosis. However, the downstream signaling pathways through which CD22 modulates microglial function, particularly during Aβ clearance, remain poorly understood.

Here, we investigated the role of CD22 in microglial Aβ handling and its underlying mechanisms. We show that CD22 is upregulated in the hippocampus of AD patients and AD model mice and can be induced by Aβ in microglia. Knocking down *Cd22* enhanced Aβ clearance in primary microglia without detectably affecting initial Aβ uptake under our conditions. Transcriptomic and biochemical analyses linked *Cd22* deficiency to downregulation of INPP5D and increased AKT phosphorylation. Furthermore, overexpressing INPP5D attenuated the enhanced Aβ clearance phenotype caused by *Cd22* knockdown. These findings support a model in which the CD22-INPP5D axis regulates microglial Aβ processing.

## 2 Materials and methods

### 2.1 Bioinformatics analysis

Postmortem transcriptomic data from the human hippocampus were obtained from the Gene Expression Omnibus (GEO; https://www.ncbi.nlm.nih.gov/geo/), specifically dataset GSE173955. This dataset comprises bulk RNA-sequencing gene expression profiles generated on the Illumina HiSeq 1,500 platform (GPL18460) and includes samples from Alzheimer's disease (AD) patients and cognitively normal control individuals. The normalized expression level of *CD22* was compared between AD and control groups. Group differences were visualized using bar plots showing mean ± SD and evaluated by two-tailed Student's *t*-test.

### 2.2 Animal samples

Archived hippocampal tissues were obtained from APP/PS1 (APPswe/PSEN1dE9) transgenic mice and age-matched wild-type littermates (6 and 9 months old) maintained in our laboratory. Mice were anesthetized with isoflurane and euthanized by cervical dislocation according to approved institutional protocols. Brains were rapidly removed, and the hippocampi were carefully dissected, collected, and stored until subsequent quantitative PCR and western blot analyses. All animal procedures were approved by the Animal Care and Use Committee of Jinfeng Laboratory.

### 2.3 Isolation and culture of primary microglia

Primary microglia were isolated from the cerebral cortices of postnatal day 0–3 C57BL/6J neonatal mice using an adapted mixed glial culture method (Li et al., 2025). Briefly, neonatal mice were anesthetized with 3% isoflurane and euthanized by cervical dislocation. Cerebral cortices were then collected under sterile conditions, and the meninges were carefully removed. The tissue was dissociated into a single-cell suspension in Dulbecco's Modified Eagle Medium (DMEM; Gibco, #11965092) supplemented with 10% fetal bovine serum (FBS; Gibco, #10437028) and 1% penicillin-streptomycin (Gibco, #15140122).

Cells were plated onto poly-D-lysine (Sigma-Aldrich, #P7280) coated T75 culture flasks and maintained at 37°C in a humidified atmosphere with 5% CO_2_. Culture medium was replaced every 2–3 days until a confluent glial layer was established. After 10–14 days *in vitro*, microglia were released from the adherent astrocytic layer by mild orbital agitation. The cell suspension was collected and centrifuged at 300 × g for 5 min. Pelleted cells were resuspended in microglia maintenance medium and seeded onto coated plates for subsequent experiments.

### 2.4 Aβ_42_ treatment

Aggregation of Aβ_1 − 42_ was performed according to a previously established protocol (Stine et al., 2011). Briefly, lyophilized Amyloid β-Peptide (1–42) (human) (Aβ_42_; Beyotime, #P9001) was first dissolved in hexafluoroisopropanol (HFIP; Beyotime, #Y063651) to eliminate pre-existing aggregates. After solvent evaporation, the resulting peptide film was reconstituted in dimethyl sulfoxide (DMSO; Sigma, #D8418) and subsequently diluted into serum-free medium to promote oligomer formation. Primary microglia were treated with vehicle control (DMSO) or 2 μM Aβ_42_ in serum-free DMEM for the indicated durations.

### 2.5 siRNA design and transfection

Small interfering RNA (siRNA) targeting mouse *Cd22* (siCd22) was synthesized by Qingke Biotechnology (mouse *Cd22* siRNA: GCAATGGATGACACCGTTAGT). A non-targeting scrambled siRNA was used as the negative control (Ctrl siRNA). For transfection experiments, primary microglia were transfected using Lipofectamine™ 3,000 (Thermo Fisher Scientific, #L3000150) diluted in Opti-MEM reduced-serum medium (Gibco, #31985070) according to the manufacturer's instructions.

### 2.6 Lentivirus production

Short hairpin RNA (shRNA) targeting mouse *Cd22* (TRCN0000067943) was selected from the RNAi Consortium (TRC) shRNA library and cloned into the pLKO.1 lentiviral vector. A non-targeting scrambled shRNA (Ctrl) cloned into the same pLKO.1 vector was used as the negative control. A plasmid encoding the full-length mouse *Inpp5d* open reading frame (ORF) was purchased from YouBio (#G132776) and subsequently subcloned into the FUW-tetO-LoxP lentiviral expression vector to generate FUW-INPP5D. Lentiviral particles were generated in HEK293FT cells with minor adaptations from previously reported protocols (Zhu et al., 2023). Briefly, HEK293FT cells were plated at a density of 5 × 106 cells per 10-cm dish and cultured overnight to reach 90% confluency. Cell co-transfection was performed using the packaging plasmids psPAX2 and pMD2.G, together with transfer plasmid (pLKO.1 or FUW-based constructs), employing Lipofectamine™ 3,000 according to the manufacturer's instructions. The transfection medium was replaced 16 h later to eliminate residual reagents. Viral-containing supernatants were harvested at 24, 40, and 48 h after transfection, pooled, and centrifuged at 300 × g for 5 min to remove cell debris. The supernatants were subsequently passed through a 0.45 μm PVDF syringe filter (Merck, #SLHVR33RS). Viral titers were quantified using an HIV-1 p24 ELISA kit (Abcam, #ab218268). Lentiviral stocks were aliquoted and stored at −80°C, and were used either immediately or after a single freeze-thaw cycle for subsequent experiments.

### 2.7 Lentiviral transduction and INPP5D rescue

Primary microglia were seeded into 6-well plates and cultured overnight. Cells were then transduced with control lentivirus, shCd22 lentivirus, FUW-INPP5D lentivirus, or the combination of shCd22 and FUW-INPP5D lentiviruses at an MOI of 10 for each virus in the presence of 8 μg/mL polybrene (Sigma, #H9268). After 16 h of incubation, the virus-containing medium was removed, and cells were washed three times with fresh medium before being cultured in complete DMEM. Cells were maintained for a total of 72 h after transduction before subsequent functional assays. INPP5D overexpression was confirmed by western blot analysis.

### 2.8 Western blot

Tissues and primary microglia were homogenized in RIPA lysis buffer (Beyotime, #P0013B) supplemented with a protease inhibitor cocktail (MCE, #HY-K0010). Total protein concentrations were measured using a bicinchoninic acid (BCA) protein assay kit (Dingguo, #BCA01). Equal amounts of protein were resolved by SDS-PAGE, using 8% polyacrylamide gels for most targets and 15% gels for Aβ, and subsequently transferred onto polyvinylidene difluoride (PVDF) membranes (Millipore, #IPVH00010). Membranes were blocked and then incubated at 4°C overnight with primary antibodies against CD22 (Proteintech, #21894-1-AP), full-length APP (Sigma, #A8717), β-Amyloid (Cell Signaling Technology, #8243), phosphorylated AKT (Proteintech, #66444-1-Ig), total AKT (Proteintech, #10176-2-AP), INPP5D (Proteintech, #19694-1-AP), and GAPDH (Proteintech, #60004-1-Ig). After extensive washing, membranes were incubated for 1 h at room temperature with horseradish peroxidase-conjugated anti-rabbit (Proteintech, #SA00001-2) or anti-mouse (Proteintech, #SA00001-1) secondary antibodies. Immunoreactive bands were detected using an enhanced chemiluminescence (ECL) substrate (Beyotime, #P0018S) and imaged with a Fusion FX5 imaging system (Vilber Lourmat, France). Band intensities were quantified using ImageJ software, and protein expression levels were normalized to GAPDH.

### 2.9 ELISA analysis of Aβ uptake and degradation

Primary microglia transduced with control lentivirus (Ctrl), shCd22, Inpp5d-overexpressing lentivirus, or their combination were cultured for 72 h prior to Aβ_42_ treatment. For Aβ_42_ uptake and degradation assays, microglia were incubated with 2 μM Aβ_42_ in serum-free DMEM for 3 h (*t* = 0 h), followed by extensive washing with PBS to remove unbound and surface-associated Aβ_42_, and further incubation in Aβ_42_-free, serum-free DMEM for an additional 3 h (*t* = 3 h), as previously described (Teng et al., 2026). Cells were then lysed and centrifuged at 12,000 × g for 15 min at 4°C. The resulting supernatants were collected for quantification of intracellular Aβ_42_ levels using an ELISA assay (Aβ_42_ ELISA kit, #E-EL-H0543c). Aβ_42_ degradation efficiency was calculated as the percentage of intracellular Aβ_42_ remaining at *t* = 3 h relative to the amount measured at *t* = 0 h.

### 2.10 Real-time quantitative PCR

Total RNA was extracted using TRIzol reagent (Sangon, #B511311) and reverse-transcribed into cDNA using a HiScript^®^ IV All-in-One Ultra RT SuperMix (Vazyme, cat. no. R43301) according to the manufacturer's instructions. Quantitative PCR was performed using gene-specific primers with a SYBR Green-based assay (Vazyme, cat. no. Q111-03) on a CFX Opus 96 Real-Time PCR Detection System (Bio-Rad). Cycle threshold (Ct) values were obtained for each reaction, and target gene expression was normalized to the endogenous control *Gapdh*. Relative mRNA expression levels were calculated using the comparative 2^−ΔΔCt^ method. All reactions were performed in triplicate from three independent experiments, and primer sequences are provided in Supplementary Table 1.

### 2.11 Bulk RNA-sequencing (RNA-seq) analysis

Total RNA was isolated from primary microglia transduced with either shCd22-expressing or control lentiviruses using TRIzol reagent. Bulk RNA-seq was performed using three independent biological replicates per group. Strand-specific RNA-seq libraries were constructed with the TruSeq Stranded RNA Library Prep Kit and sequenced using the NovaSeq 6,000 SP Reagent Kit on an Illumina NovaSeq 6,000 platform to generate 50-bp paired-end reads. All sequencing procedures were performed at the Jinfeng Laboratory Single-Cell Sequencing Center.

Raw sequencing reads were processed using Cutadapt (version 4.7) to remove low-quality bases at the 5′ end and to filter out reads with mapping quality scores below 20. Cleaned reads were then aligned to the reference genome using HISAT2 (Galaxy version 2.2.1). Gene-level read counts were quantified with featureCounts (Subread version 2.0.6) based on annotated genomic features. Differential expression analysis was performed in R using the DESeq2 package (version 1.42.1), with normalized read counts used for statistical testing. Genes with an adjusted *P* value <0.05 and an absolute log2 fold change greater than 1 were considered differentially expressed. Volcano plots were generated using the ggplot2 package (version 3.5.0). Functional enrichment analysis of differentially expressed genes was conducted using the clusterProfiler package (version 4.10.1).

### 2.12 Statistical analysis

Sample sizes were determined based on established field standards and prior publications. Quantitative data are presented as mean ± standard deviation (SD). For comparisons between two groups, unpaired two-tailed Student's *t* tests were used. For experiments involving more than two groups, one-way or two-way analysis of variance (ANOVA) was used as appropriate, followed by *post hoc* multiple-comparisons tests as indicated in the figure legends. Statistical significance was defined as *P* < 0.05. No formal outlier detection was performed, and all data points were retained for analysis. Statistical analyses were conducted using GraphPad Prism version 8.0.

## 3 Results

### 3.1 *CD22* is upregulated in AD patients and transgenic mouse models and is induced by Aβ in primary microglia

**Figure 1:**
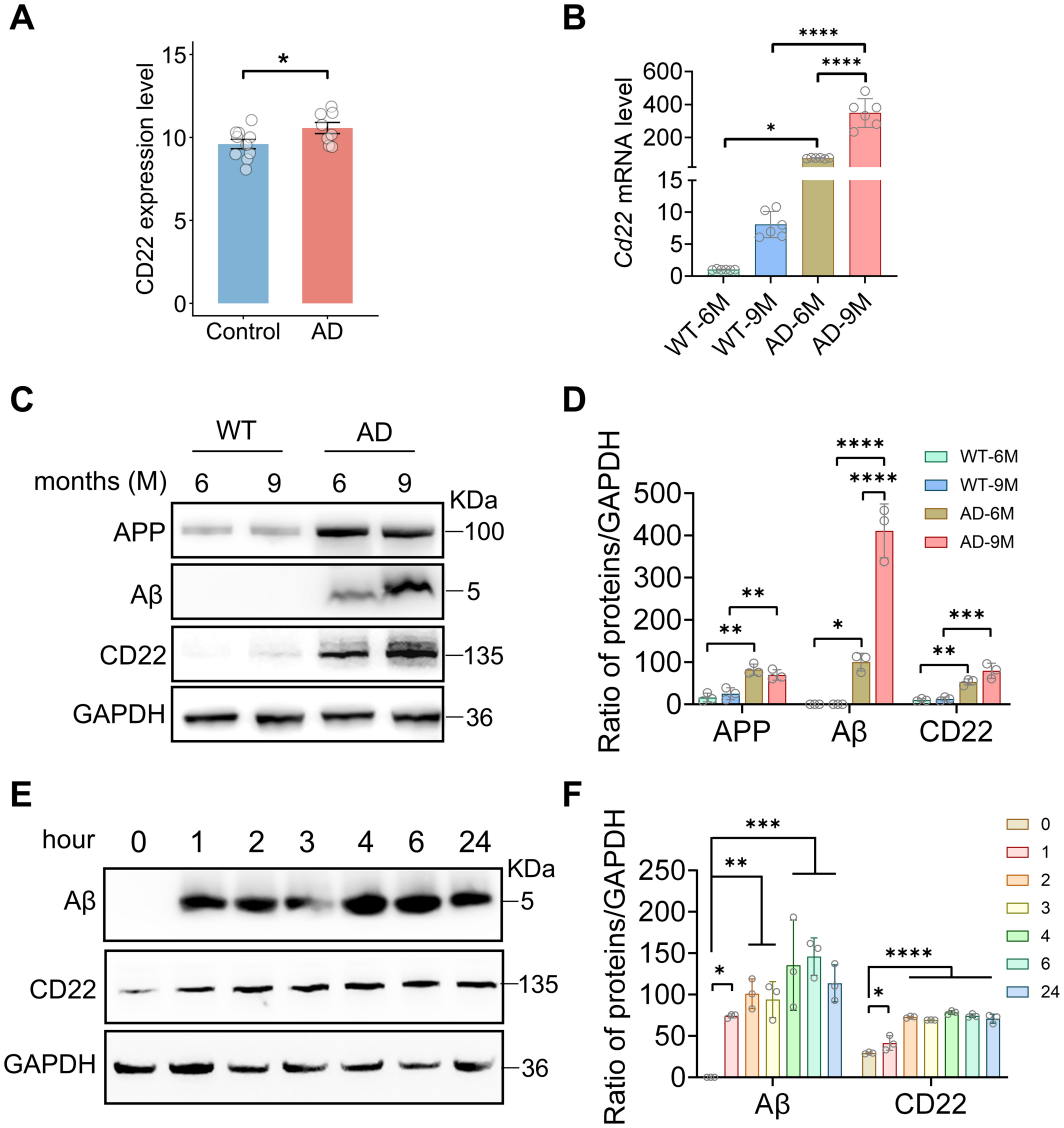
CD22 is upregulated in AD patients and AD transgenic mice and is induced by Aβ in primary microglia. **(A)** Normalized *CD22* expression in the hippocampus of AD patients (*n* = 8) and cognitively normal controls (*n* = 10) from the GSE173955 RNA-seq dataset. **(B)** Relative *Cd22* mRNA expression in the hippocampus of APP/PS1 transgenic mice and age-matched wild-type littermates at 6 and 9 months of age (*n* = 6 per group). **(C)** Representative Western blots of APP, Aβ, and CD22 protein levels in hippocampal tissues from WT and AD mice at 6 and 9 months. GAPDH served as a loading control. **(D)** Quantification of protein levels normalized to GAPDH. **(E)** Primary microglia were treated with 2 μM Aβ_42_ for 0–24 h. Representative Western blots of Aβ and CD22 protein levels. **(F)** Quantification of protein levels normalized to GAPDH. Data are presented as mean ± SD. Statistical significance was determined by unpaired two-tailed *t*-test **(A)**, one-way ANOVA with Dunnett's multiple comparisons vs. 0 h **(F)**, and two-way ANOVA with Sidak's multiple comparisons (**B, D**). * *P* < 0.05, ** *P* < 0.01, *** *P* < 0.001, **** *P* < 0.0001.

To explore the relevance of CD22 in AD, we examined its expression in a publicly available human hippocampal RNA-seq dataset (GSE173955). *CD22* expression was significantly elevated in the hippocampus of AD patients compared with cognitively normal controls (Figure 1A, *P* = 0.0437), indicating disease-associated upregulation of CD22 in the AD hippocampus. To validate these findings *in vivo*, we examined *Cd22* expression in AD transgenic mice. qPCR analysis revealed significantly higher *Cd22* mRNA levels in the hippocampus of AD mice compared with age-matched wild-type (WT) controls at both 6 and 9 months (Figure 1B). Moreover, *Cd22* expression increased modestly with age in WT mice, whereas a more pronounced increase was observed in AD mice. Western blot analysis confirmed markedly elevated CD22 protein levels in AD mice at both 6 and 9 months (Figure 1C). Quantification normalized to GAPDH (Figure 1D) showed significantly higher CD22 in AD versus WT mice at both time points, with the greatest increase at 9 months. Aβ levels were also significantly elevated in AD mice, especially at 9 months, consistent with progressive amyloid accumulation. Notably, the progression of CD22 upregulation in AD mice coincided with the increasing burden of Aβ, suggesting a potential association between CD22 expression and amyloid pathology.

To assess direct regulation of CD22 by Aβ, primary microglia were treated with 2 μM Aβ_42_ for the indicated times. Western blot analysis revealed that Aβ_42_ exposure induced a rapid and sustained increase in CD22 protein levels (Figure 1E). Quantification showed that CD22 expression was significantly upregulated as early as 1 h after Aβ_42_ treatment, peaked at 4-6 h, and remained elevated above baseline at 24 h (Figure 1F). In contrast, the levels of exogenously applied Aβ remained stably detectable throughout the 24-h treatment period. These data indicate that Aβ is sufficient to directly induce CD22 expression in microglia. Although CD22 expression reached its maximum at 4–6 h, it remained markedly elevated after 24 h of Aβ exposure. Therefore, the 24-h time point was selected for subsequent functional experiments to assess the cumulative effect of *Cd22* knockdown on intracellular Aβ processing.

### 3.2 *Cd22* knockdown enhances Aβ clearance in primary microglia

**Figure 2:**
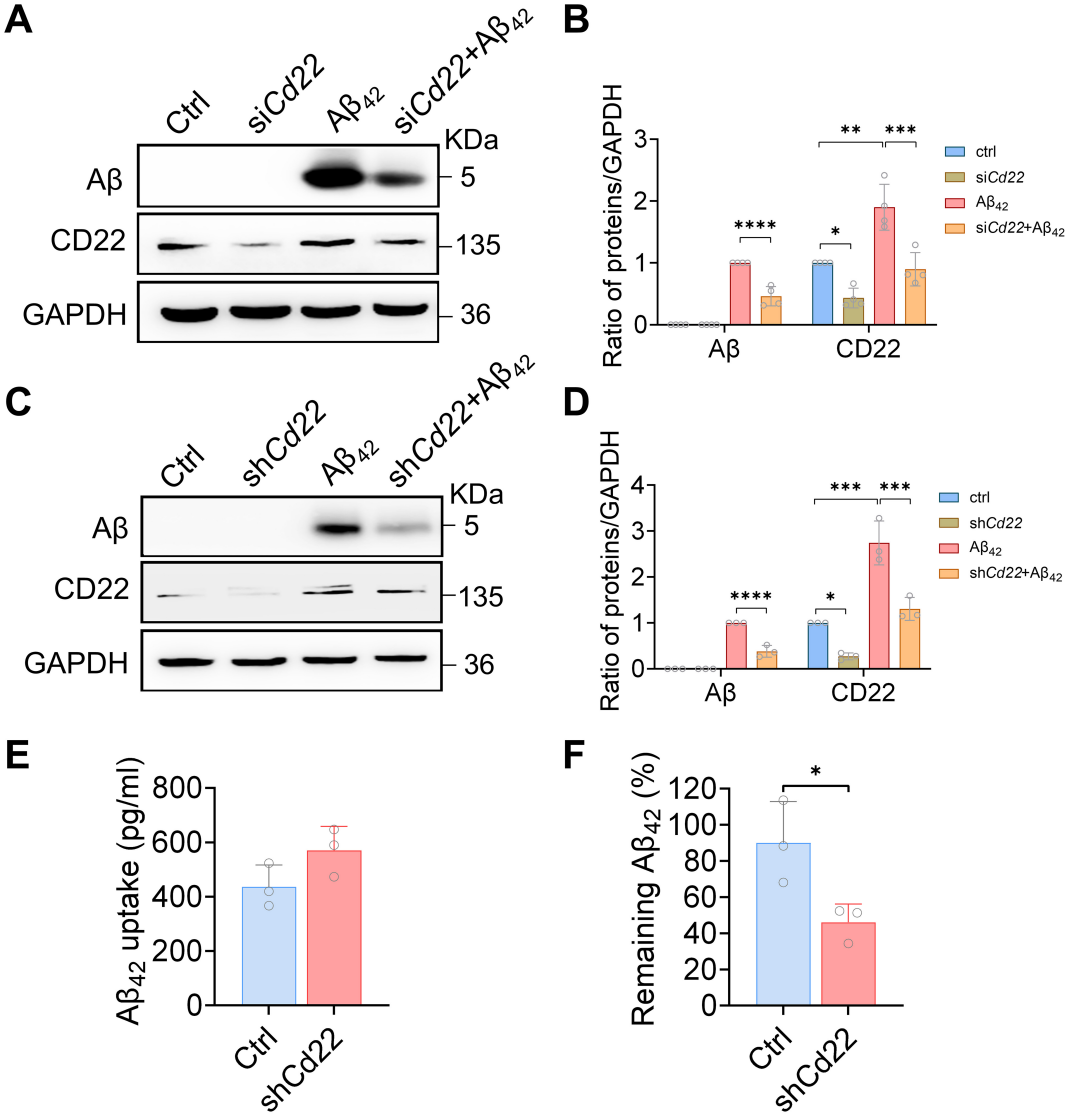
*Cd22* knockdown enhances microglial Aβ clearance. **(A)** Representative Western blots showing Aβ and CD22 expression in primary microglia transfected with control siRNA (Ctrl) or *Cd22* siRNA (siCd22) for 72 h, followed by Aβ_42_ treatment for 24 h. **(B)** Quantification of protein levels normalized to GAPDH and expressed relative to the Ctrl group, which was set to 1. **(C)** Representative Western blots showing Aβ and CD22 expression in primary microglia infected with lentivirus expressing control (Ctrl) or *Cd22* shRNA (shCd22) for 72 h, followed by Aβ_42_ treatment for 24 h. **(D)** Quantification of protein levels normalized to GAPDH and expressed relative to the Ctrl group, which was set to 1. **(E)** Intracellular Aβ_42_ levels in control and shCd22-treated microglia were quantified using an Aβ_42_ ELISA assay immediately after Aβ_42_ exposure (*t* = 0 h), reflecting Aβ_42_ uptake. **(F)** Intracellular Aβ_42_ remaining in control and shCd22-treated primary microglia at 3 h after extracellular Aβ_42_ removal, normalized to *t* = 0 h levels. Data are mean ± SD. Statistical analysis was performed using one-way ANOVA followed by Tukey's multiple comparisons **(B, D)** and unpaired two-tailed Student's *t*-test (**E, F**). *P < 0.05, **P < 0.01, ***P < 0.001, ****P < 0.0001.

Having observed CD22 upregulation in AD mice and Aβ-stimulated microglia, we next asked whether CD22 influences microglial handling of Aβ. Microglia were transfected with *Cd22* siRNA (siCd22) and then exposed to Aβ_42_. Western blot confirmed efficient *Cd22* knockdown (Figure 2A). Silencing of *Cd22* significantly reduced intracellular Aβ levels after Aβ_42_ treatment compared with control siRNA-transfected cells (Figures 2A, B). To exclude off-target effects, we also performed lentivirus-mediated *Cd22* shRNA knockdown (shCd22). Consistent with the siRNA results, shCd22 markedly decreased intracellular Aβ accumulation following Aβ_42_ treatment (Figures 2C, D), confirming that the effect was due to *Cd22* knockdown. To determine whether CD22 influences Aβ uptake, intracellular levels of Aβ_42_ were measured immediately after exposure (*t* = 0 h). *Cd22* knockdown did not significantly affect Aβ_42_ uptake relative to control cells (Figure 2E). However, at 3 h after removal of extracellular Aβ_42_, *Cd22*-deficient cells exhibited a significantly lower level of intracellular Aβ_42_ remaining after the clearance period (Figure 2F), indicating enhanced intracellular Aβ degradation. Taken together, these data suggest that *Cd22* knockdown enhances intracellular degradation or clearance of Aβ rather than impairing its uptake.

### 3.3 Transcriptomic profiling identifies Inpp5d as a potential downstream effector of Cd22

**Figure 3:**
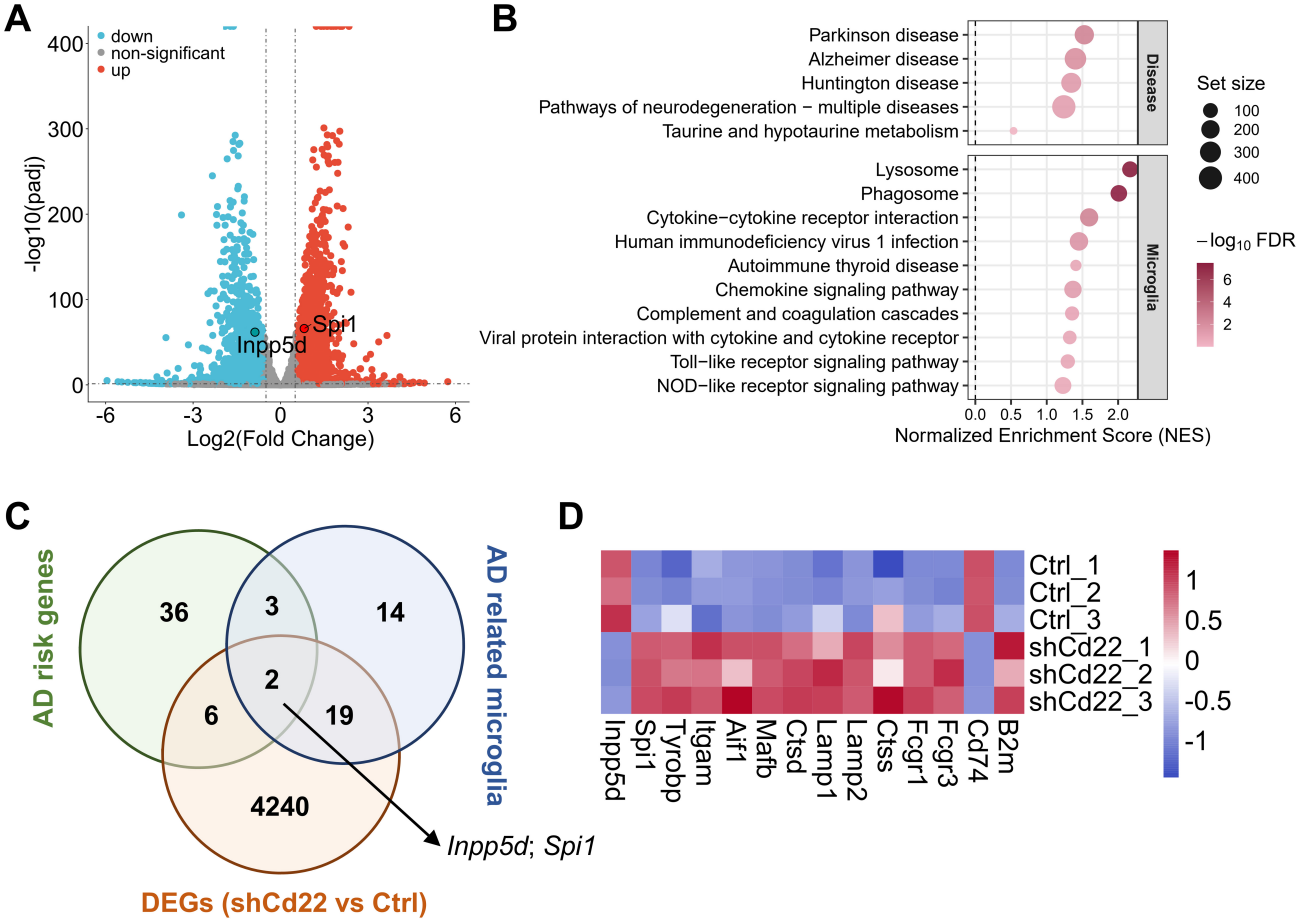
Transcriptomic profiling identifies *Cd22*-associated changes in microglial functional pathways **(A)** Volcano plot of differentially expressed genes (DEGs) in *Cd22*-knockdown primary microglia vs. controls, showing 2,068 downregulated and 2,199 upregulated genes. **(B)** KEGG-based gene set enrichment analysis (GSEA) of pathways enriched in *Cd22*-knockdown microglia, ranked by normalized enrichment score (NES; positive NES indicates enrichment among upregulated genes). **(C)** Venn diagram of overlap between DEGs, literature-curated AD risk genes, and AD-associated microglial signature genes. *Inpp5d* and *Spi1* were identified as shared candidates. **(D)** Heatmap of selected genes related to AD-associated microglial signaling, lysosomal function, and phagocytic/antigen-processing pathways in control and *Cd22*-knockdown microglia. Values represent row-wise z-score RNA-seq expression levels (red = higher than gene mean; blue = lower than gene mean).

To understand how CD22 regulates microglial Aβ clearance, we performed bulk RNA sequencing (bulk RNA-seq) on primary microglia after *Cd22* knockdown. Differential expression analysis revealed extensive transcriptional reprogramming, with 2,068 downregulated and 2,199 upregulated genes compared to control microglia (Figure 3A; Supplementary Table 2), indicating that CD22 broadly regulates microglial gene expression. To characterize the functional impact of *Cd22* depletion, we conducted KEGG-based gene set enrichment analysis (GSEA). Pathways linked to neurodegenerative diseases, lysosomal function, cytokine signaling, and phagocytosis were significantly enriched (Figure 3B). Representative enrichment plots confirmed positive enrichment of Fcγ receptor-mediated phagocytosis, antigen processing and presentation, and lysosomal pathways in *Cd22*-deficient cells (Supplementary Figure 1). These pathways are closely associated with microglial Aβ handling and are consistent with the enhanced Aβ clearance observed following *Cd22* knockdown, suggesting a coordinated transcriptional shift toward increased degradative and immune activity.

To identify disease-relevant mediators connecting these pathway changes to AD, we intersected the DEGs with literature-curated sets of representative AD risk genes and AD-associated microglial genes (Keren-Shaul et al., 2017; Bellenguez et al., 2022). This analysis revealed two shared candidates, *Inpp5d* and *Spi1* (Figure 3C; Supplementary Table 3), with *Inpp5d* prioritized for further validation due to its established role in AD-associated microglial signaling (Samuels et al., 2023; Terzioglu and Young-Pearse, 2023). A heatmap of representative genes showed coordinated upregulation of pathways related to microglial activation, lysosomal function, and phagocytic/antigen-processing processes in *Cd22* knockdown cells (Figure 3D). INPP5D expression was reduced, further supporting its potential role as a downstream mediator of CD22 signaling.

### 3.4 *Cd22* knockdown suppresses Inpp5d expression in microglia

**Figure 4:**
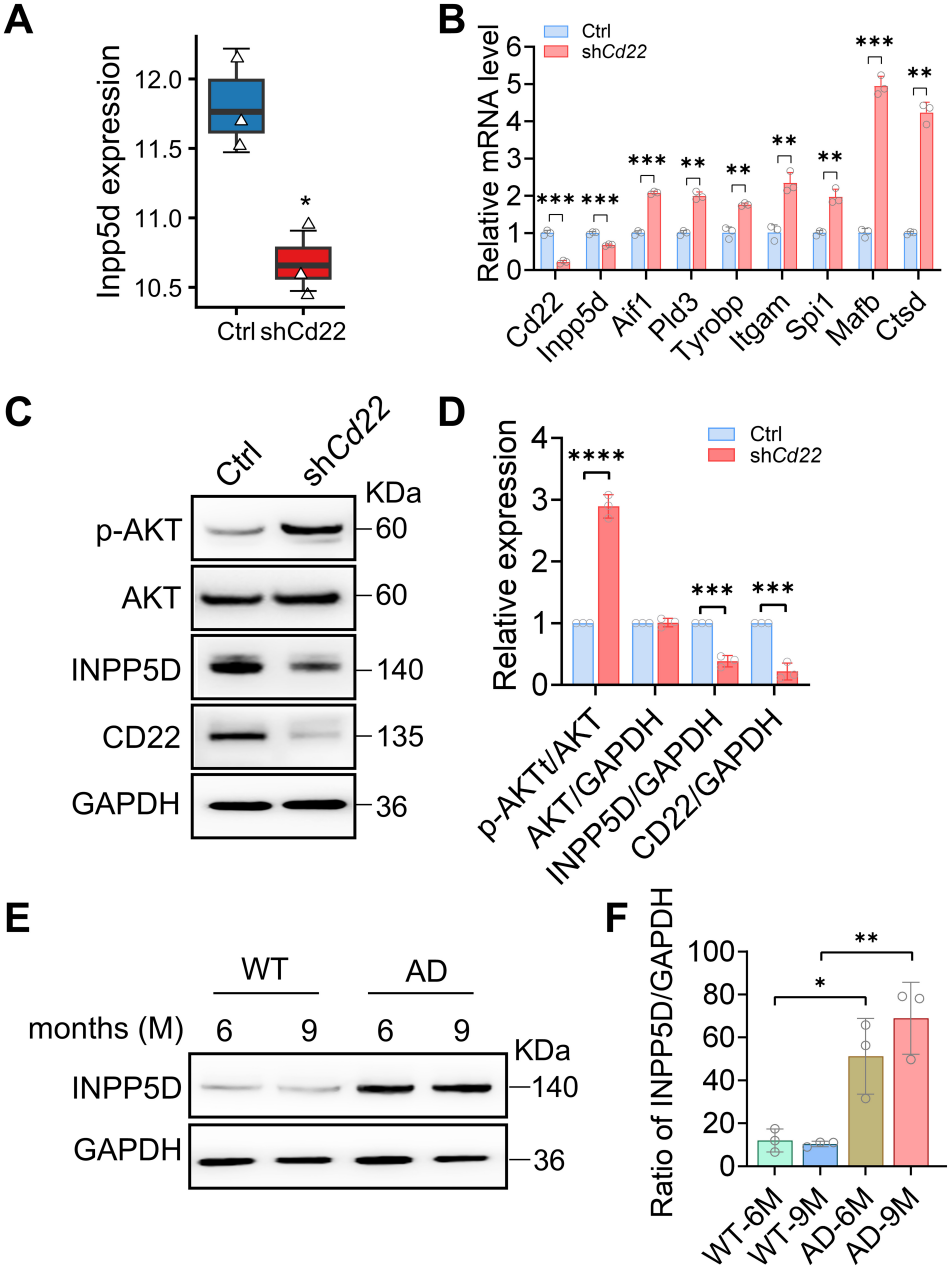
*Cd22* knockdown suppresses Inpp5d expression in primary microglia and INPP5D is elevated in APP/PS1 mouse hippocampus. **(A)** Normalized RNA-seq expression levels of Inpp5d in control and *Cd22*-knockdown primary microglia. **(B)** qPCR analysis of *Cd22, Inpp5d*, and selected microglia-associated genes. mRNA levels were normalized to *Gapdh* and expressed relative to the Ctrl group, which was set to 1. **(C)** Representative Western blot images showing INPP5D, phosphorylated AKT (p-AKT), total AKT, and CD22 protein levels in control and *Cd22*-knockdown cells. GAPDH was used as a loading control. **(D)** Quantification of protein levels normalized to GAPDH (INPP5D and CD22) or total AKT (p-AKT), expressed relative to the Ctrl group, which was set to 1. **(E)** Representative Western blots showing INPP5D protein levels in hippocampal tissues from APP/PS1 transgenic mice and age-matched wild-type littermates at 6 and 9 months of age. GAPDH served as a loading control. **(F)** Quantification of INPP5D protein levels normalized to GAPDH. Data are presented as mean ± SD. Statistical significance was determined by unpaired two-tailed Student's *t*-test (**A, B, D**). *P < 0.05, **P < 0.01, ***P < 0.001, ****P < 0.0001.

Having identified INPP5D as a candidate downstream mediator via transcriptomic profiling, we next assessed whether CD22 regulates INPP5D at transcriptional and protein levels. RNA-seq revealed a significant decrease in *Inpp5d* expression following *Cd22* knockdown (Figure 4A). Quantitative PCR further confirmed that silencing *Cd22* markedly reduced *Inpp5d* mRNA levels in microglia (Figure 4B). Additionally, several microglia-associated genes were significantly altered upon *Cd22* suppression, consistent with the broader transcriptional reprogramming observed in the RNA-seq dataset (Figure 4B).

To determine if CD22 regulates INPP5D at the protein level and modulates downstream signaling, we performed Western blot analysis. *Cd22* knockdown significantly reduced INPP5D protein expression compared with control microglia (Figures 4C, D). INPP5D suppression was accompanied by a marked increase in phosphorylated AKT (p-AKT) levels, while total AKT levels remained unchanged (Figures 4C, D). Overall, these results support a model in which INPP5D acts as a downstream effector linking CD22 to AKT-mediated signaling.

To further assess the relevance of INPP5D *in vivo*, we examined INPP5D protein expression in hippocampal tissues from APP/PS1 transgenic mice and age-matched wild-type littermates. Western blot analysis demonstrated that INPP5D protein levels were significantly increased in APP/PS1 mice compared with WT controls (Figures 4E, F). These findings are consistent with previous reports of elevated INPP5D expression in AD brains and provide additional *in vivo* evidence that INPP5D is dysregulated in AD.

### 3.5 INPP5D overexpression attenuates the enhanced Aβ clearance induced by *Cd22* knockdown

**Figure 5:**
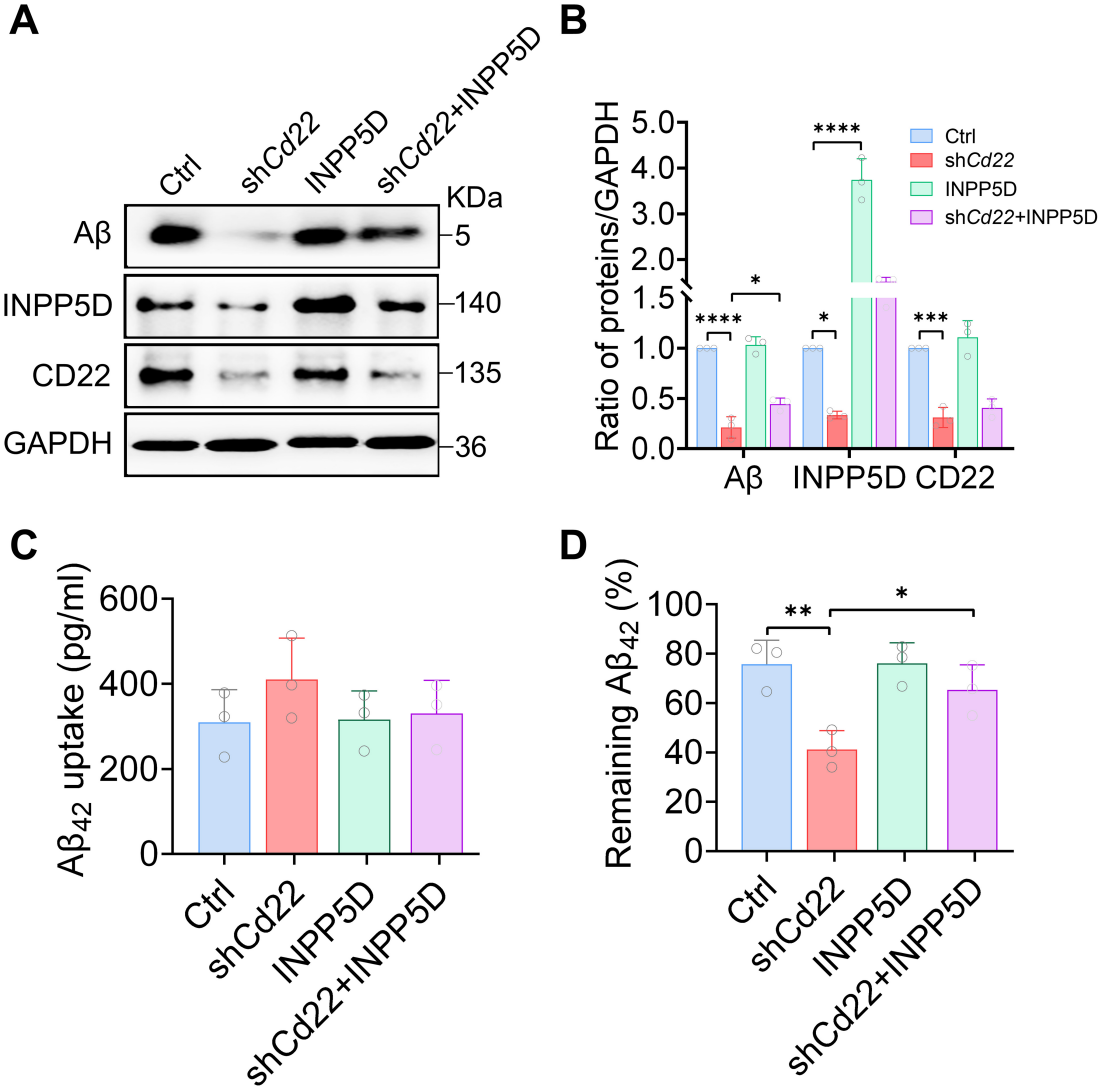
INPP5D overexpression attenuates *Cd22* knockdown-mediated enhancement of Aβ clearance. **(A)** Representative Western blot images showing *Cd22* knockdown, INPP5D overexpression, and intracellular Aβ_42_ levels under the indicated conditions. **(B)** Densitometric quantification of protein levels normalized to GAPDH and expressed relative to the Ctrl group, which was set to 1. **(C)** Quantification of intracellular Aβ_42_ uptake at *t* = 0 h in control (Ctrl), *Cd22* knockdown (shCd22), INPP5D overexpression (INPP5D), and combined *Cd22* knockdown + INPP5D overexpression (shCd22 + INPP5D) conditions. **(D)** Quantification of intracellular Aβ_42_ remaining at 3 h after Aβ_42_ removal, normalized to *t* = 0 h. Data are presented as mean ± SD. Statistical significance was determined by one-way ANOVA followed by Tukey's multiple comparisons (**B, C, D**). ^*^P < 0.05, ^**^P < 0.01, ^***^P < 0.001, ^****^P < 0.0001.

To determine whether INPP5D mediates CD22′s regulation of microglial Aβ clearance, we performed rescue experiments by overexpressing INPP5D in *Cd22*-knockdown microglia. Western blot analysis confirmed efficient *Cd22* knockdown and robust INPP5D overexpression in the corresponding experimental groups (Figure 5A), which was further verified by densitometric quantification (Figure 5B).

Following validation of the experimental manipulations, we evaluated Aβ_42_ uptake and intracellular clearance. Consistent with our previous findings, *Cd22* knockdown did not significantly affect Aβ_42_ uptake compared with control cells (Figure 5C). Likewise, INPP5D overexpression alone or in combination with *Cd22* knockdown did not significantly alter Aβ_42_ uptake, indicating that modulation of the CD22-INPP5D axis does not influence the initial internalization of Aβ_42_.

In contrast, *Cd22* knockdown significantly reduced the proportion of intracellular Aβ_42_ remaining after the degradation period, indicating enhanced intracellular Aβ_42_ clearance (Figure 5D). Importantly, INPP5D overexpression in *Cd22*-deficient microglia partially restored the amount of intracellular Aβ_42_ remaining toward control levels, demonstrating a partial rescue of the enhanced clearance phenotype. INPP5D overexpression alone did not significantly affect Aβ_42_ degradation. These findings indicate that INPP5D functions as a downstream effector that partially mediates the regulatory effect of CD22 on microglial Aβ clearance.

## 4 Discussion

**Figure 6:**
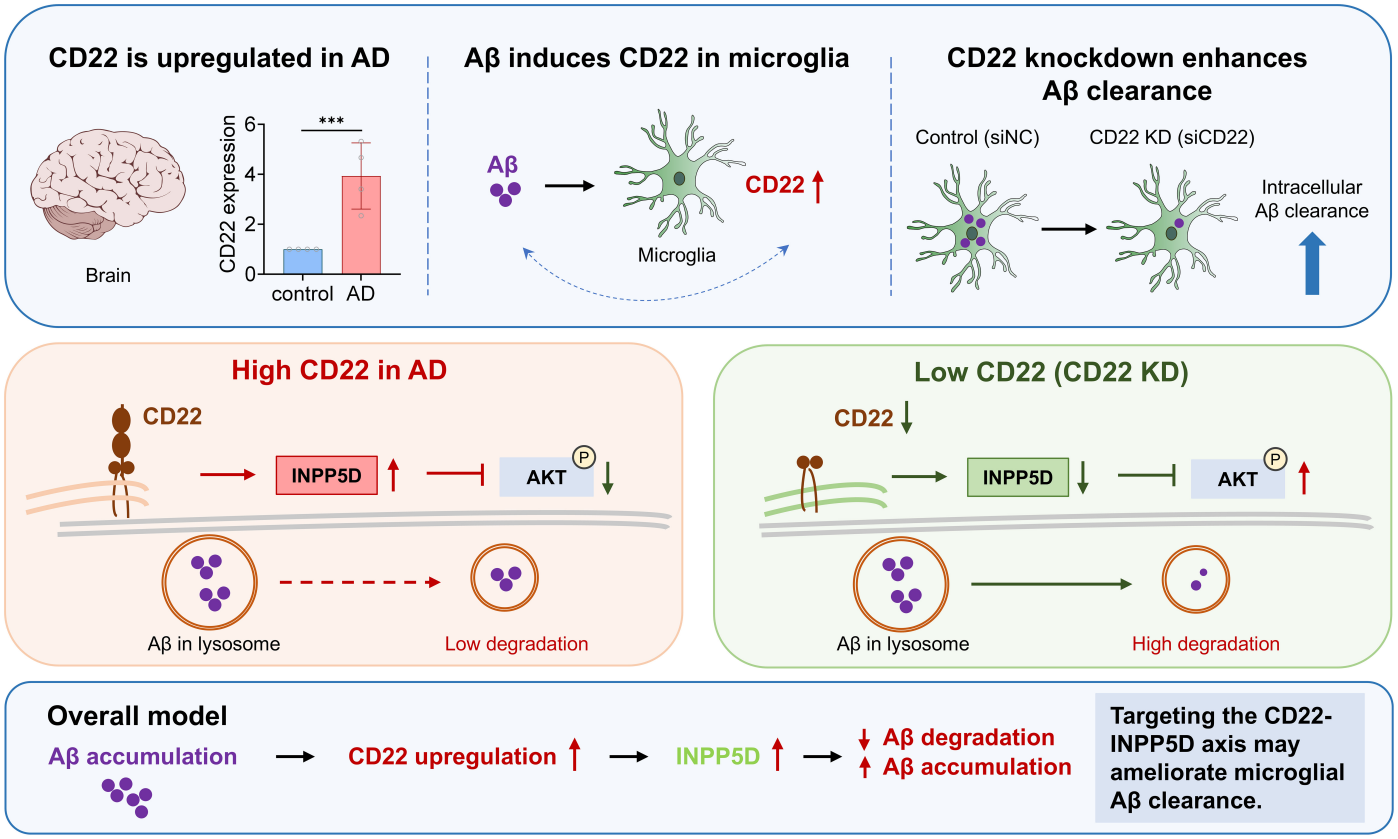
Proposed model of CD22-INPP5D signaling in the regulation of intracellular Aβ clearance in microglia. CD22 expression is elevated in the hippocampus of Alzheimer's disease (AD) and is induced by Aβ stimulation in primary microglia. Increased CD22 expression is associated with elevated INPP5D expression through an as-yet undefined mechanism. Consistent with the inhibitory role of INPP5D in AKT signaling, high CD22 levels are accompanied by reduced AKT phosphorylation and impaired intracellular Aβ clearance, resulting in increased intracellular Aβ retention. Conversely, *Cd22* knockdown decreases INPP5D expression, is accompanied by increased AKT phosphorylation, and enhances intracellular Aβ clearance without affecting initial Aβ uptake. Overexpression of INPP5D partially attenuates the enhanced Aβ clearance induced by *Cd22* knockdown, supporting INPP5D as a functional downstream effector of CD22. Because the direct mechanistic relationship between altered AKT phosphorylation and enhanced intracellular Aβ clearance was not established in the present study, the association between these events is presented as a proposed model. Overall, these findings suggest that the CD22-INPP5D axis may represent a potential therapeutic target for enhancing microglial Aβ clearance in AD.

Our findings support a working model (Figure 6) in which CD22 restrains microglial Aβ clearance primarily by suppressing intracellular degradation rather than initial uptake. The enhanced clearance following *Cd22* knockdown, coupled with transcriptomic enrichment of lysosomal and phagocytic pathways, functionally distinguishes Aβ uptake from its intracellular processing. This positions CD22 as a selective regulator of microglial degradation efficiency, refining the current understanding of Aβ handling. Microglial dysfunction is central to AD pathogenesis. While protective at early stages, chronic Aβ exposure ultimately impairs phagocytic capacity, contributing to amyloid accumulation (Miao et al., 2023; Hassan et al., 2025). Multiple AD risk genes identified by GWAS are highly enriched in microglia and converge on immune receptor signaling pathways. Among them, INPP5D negatively regulates AKT signaling and is genetically linked to AD (Bellenguez et al., 2022). Elevated INPP5D expression has been reported in AD brains, but its upstream regulators remain elusive. Here, we identify CD22 as a functional upstream regulator of INPP5D, revealing a previously unrecognized association between an inhibitory receptor and a key AD risk phosphatase. These findings suggest a signaling connection between CD22 and INPP5D that may contribute to microglial dysfunction in AD.

CD22, a known inhibitory receptor in B cells, has recently been identified in aging mouse microglia, where it suppresses debris clearance (Pluvinage et al., 2019; Bu et al., 2022). Our work extends this observation to AD pathology. An important consideration is the apparent difference in CD22 expression between mouse and human brains. Pluvinage et al. (2019) demonstrated functional CD22 expression in mouse microglia and showed that CD22 blockade restores microglial phagocytosis during aging. In contrast, subsequent analyses of human brain tissue suggested that CD22 expression is enriched in oligodendrocytes rather than microglia (Pluvinage et al., 2021). Notably, the same study also identified IGF2R as a functional binding partner of CD22 that regulates lysosomal function (Pluvinage et al., 2021). Whether CD22-IGF2R signaling also contributes to microglial intracellular Aβ degradation in AD remains unknown and warrants further investigation. These findings suggest potential species-specific differences in CD22 biology. Because our mechanistic analyses were performed in primary mouse microglia, whether a similar CD22-INPP5D signaling axis operates in human microglia remains to be determined and requires further investigation using human microglial models.

We further show that Aβ_42_ itself induces CD22 expression, suggesting a feed-forward loop where amyloid accumulation upregulates CD22, further inhibiting clearance and exacerbating deposition. This provides a mechanistic basis for how Aβ can actively suppress its own clearance. Transcriptomic analysis revealed broad reprogramming of microglial gene expression following *Cd22* knockdown, with enrichment of pathways related to phagocytosis, antigen processing, and lysosomal function. These changes are consistent with enhanced degradative and immune-processing capacity (Pluvinage et al., 2019; Antignano et al., 2023). Suppression of INPP5D was accompanied by increased AKT phosphorylation, consistent with its established role as a negative regulator of PI3K signaling (Terzioglu and Young-Pearse, 2023). Rescue experiments demonstrated that enforced INPP5D expression partially reversed the enhanced Aβ clearance induced by Cd22 knockdown, supporting INPP5D as a downstream effector of CD22 signaling. Together, these findings suggest that CD22 regulates microglial Aβ clearance, at least in part, through the INPP5D-AKT signaling axis. Consistent with reduced INPP5D expression following Cd22 knockdown, AKT phosphorylation was increased and accompanied by enhanced Aβ clearance. Although these findings support a potential role for AKT signaling in mediating the effects of CD22, further mechanistic studies will be required to establish the direct regulatory relationship.

From a therapeutic perspective, our findings suggest that targeting the CD22-INPP5D signaling axis may enhance microglial Aβ clearance. Consistent with previous studies showing that CD22 blockade restores phagocytic activity in aged mouse microglia, our data indicate that reducing CD22 signaling decreases INPP5D expression and enhances intracellular Aβ degradation. Therefore, therapeutic strategies aimed at inhibiting CD22 or modulating downstream INPP5D signaling may improve microglial clearance capacity in AD. Nevertheless, the safety, efficacy, and translational relevance of such approaches require validation in human models and *in vivo* studies.

Several limitations should be considered. First, mechanistic analyses were performed in primary microglia, which may not fully recapitulate the *in vivo* state. Validation in conditional *Cd22* knockout mouse models would further strengthen the physiological relevance of this signaling axis. Second, although our data demonstrate that *Cd22* knockdown reduces INPP5D expression at both the mRNA and protein levels, the molecular mechanism underlying this regulation remains unresolved. Whether CD22 regulates INPP5D through transcriptional, post-transcriptional, or indirect signaling mechanisms requires further investigation. Moreover, CD22 may engage additional downstream pathways or signaling adaptors, including IGF2R-dependent lysosomal signaling, that contribute to microglial regulation. Future studies exploring receptor-proximal signaling complexes and phosphatase recruitment will be important for elucidating CD22-mediated signaling networks.

In summary, we identify CD22 as an Aβ-inducible brake on microglial Aβ clearance and define INPP5D as a key downstream effector associated with altered AKT signaling. By elucidating this signaling axis, which specifically constrains intracellular Aβ degradation, our work expands the current understanding of microglial dysfunction in AD and highlights the CD22-INPP5D interface as a potential therapeutic target for enhancing amyloid clearance.

## Data Availability

The datasets presented in this study can be found in online repositories. The names of the repository/repositories and accession number(s) can be found in the article/Supplementary material.
