# Supplementary material for: CD22 regulates Aβ clearance in microglia through INPP5D signaling
Source: Front Cell Neurosci. 2026 Jul 28;20:1886995. doi: 10.3389/fncel.2026.1886995 (PMC13457040; doi:10.3389/fncel.2026.1886995)
Supplement: Supplementary file 1 [file Data_Sheet_1.docx]

##### **Supplementary Information for**

**CD22 regulates Aβ clearance in microglia through INPP5D signaling**

De-Zhi Zou^1^, Jun Li^2^, Qiu-Shuang Long^3^, Ruo-Qi Zhang^3^, Bing-Lin Zhu^3,^*, Jia-Ze Tan^1,^*

^1^ Department of Neurology, The First Affiliated Hospital of Chongqing Medical University, Chongqing, 400016, China

^2^ Brain Research Center and State Key Laboratory of Trauma, Burns, and Combined Injury, the Army Medical University (Third Military Medical University), Chongqing, 400038, China

^3^ Jinfeng Laboratory, Chongqing, 401329, China

* Correspondence and requests for materials should be addressed to:
Bing-Lin Zhu (zhubinglin@jflab.ac.cn) and Jia-Ze Tan (258702855@qq.com)

**Contents**

**Figure S1. GSEA reveals activation of phagocytic and lysosomal pathways in *Cd22*-knockdown primary microglia.**

**Figure S2. Full-length, uncropped Western blot images for Figure 1C and 1E.**

**Figure S3. Full-length, uncropped Western blot images for Figure 2A and 2C.**

**Figure S4. Full-length, uncropped Western blot images for Figure 4C and 5C.**

**Table S1. Primer sequences used for quantitative real-time PCR.**

**Table S2. Differentially expressed genes (DEGs) in *Cd22* knockdown primary microglia.**

**Table S3. List of Alzheimer’s disease risk genes and microglia-associated genes.**

**Figure S1**

**GSEA reveals activation of phagocytic and lysosomal pathways in *Cd22*-knockdown primary microglia.** Representative GSEA plots showing positive enrichment of Fcγ receptor-mediated phagocytosis (A), antigen processing and presentation (B), and lysosomal pathways (C) in *Cd22*-knockdown microglia. Genes are ranked based on differential expression between *Cd22* knockdown and control conditions. Enrichment score (ES) is shown at the top, with vertical black bars indicating the positions of genes within each gene set.

**Figure S2**

**Full-length, uncropped Western blot images for Figure 1C and 1E.** Red boxes indicate how the images were cropped to produce Figure 1C and 1E.

**Figure S3**

**Full-length, uncropped Western blot images for Figure 2A and 2C.** Red boxes indicate how the images were cropped to produce Figure 2A and 2C.

**Figure S4**

**Full-length, uncropped Western blot images for Figure 4C and 5C.** Red boxes indicate how the images were cropped to produce Figure 4C and 5C.
